# Modification of Living Diatom, *Thalassiosira weissflogii*, with a Calcium Precursor through a Calcium Uptake Mechanism: A Next Generation Biomaterial for Advanced Delivery Systems

**DOI:** 10.1021/acsabm.4c00431

**Published:** 2024-05-17

**Authors:** Asrizal Abdul Rahman, Isma Liza Mohd Isa, Syed A. M. Tofail, Lukasz Bartlomiej, Brian J. Rodriguez, Manus J. Biggs, Abhay Pandit

**Affiliations:** † CÚRAM, SFI Research Centre for Medical Devices, University of Galway, Galway H91 W2TY, Ireland; ‡ Department of Anatomy, Faculty of Medicine, Universiti Kebangsaan Malaysia, 56000 Cheras, Kuala Lumpur, Malaysia; § Materials and Surface Science Institute, University of Limerick, Limerick V94 T9PX, Ireland; ∥ Conway Institute of Biomolecular and Biomedical Research and School of Physics, University College Dublin, Dublin 4, Ireland

**Keywords:** diatoms, *Thalassiosira weissflogii*, chemical modification, calcium uptake, antibody-binding delivery system

## Abstract

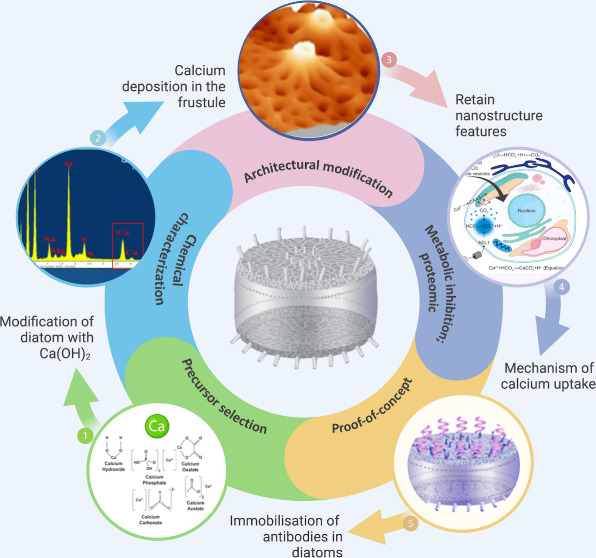

The diatom’s frustule, characterized by its rugged and porous exterior, exhibits a remarkable biomimetic morphology attributable to its highly ordered pores, extensive surface area, and unique architecture. Despite these advantages, the toxicity and nonbiodegradable nature of silica-based organisms pose a significant challenge when attempting to utilize these organisms as nanotopographically functionalized microparticles in the realm of biomedicine. In this study, we addressed this limitation by modulating the chemical composition of diatom microparticles by modulating the active silica metabolic uptake mechanism while maintaining their intricate three-dimensional architecture through calcium incorporation into living diatoms. Here, the diatom *Thalassiosira weissflogii* was chemically modified to replace its silica composition with a biodegradable calcium template, while simultaneously preserving the unique three-dimensional (3D) frustule structure with hierarchical patterns of pores and nanoscale architectural features, which was evident by the deposition of calcium as calcium carbonate. Calcium hydroxide is incorporated into the exoskeleton through the active mechanism of calcium uptake via a carbon-concentrating mechanism, without altering the microstructure. Our findings suggest that calcium-modified diatoms hold potential as a nature-inspired delivery system for immunotherapy through antibody-specific binding.

## Introduction

Diatoms comprise a family of unicellular algae with a distinctive, transparent silicon dioxide cell wall, termed the frustule, which consists of two valves exhibiting a topographical surface^1^ possessing pores and protrusions ranging from 5 to 200 μm.^2^ The sophisticated microarchitecture of this unicellular algae has attracted significant interest within the scientific community, with a view to the possible use of these nanoparticles in the development of catalysis, materials for separation science, optics, and drug delivery systems.^3,4^

Critically, most biomedical applications have focused on the inorganic chemistry and physical properties of nonviable diatoms,^5^ and silica-based templating approaches have been widely explored.^6−8^ Functionalizing diatom biosilica *in vivo* with sodium alendronate has been shown to exhibit osteoconductive properties while inhibiting osteoclasts.^9^ Cyclic nitroxide 2,6,6-tetramethylpiperidine-N-oxyl (TEMPO)-functionalized diatom biosilica demonstrated antioxidative properties and supported fibroblast viability.^10^ However, issues relating to cytotoxicity^11^ and the long-term degradability of the silica structure *in vivo* have hampered the development of diatom-derived microparticles for clinical use. Furthermore, the surface density of frustule silanol groups, which can interact with the surface of the phospholipids of red blood cell membranes, has been attributed to hemolysis *in vitro*,^12^ limiting the extent of feasible diatom-focused approaches as biomaterials *in vivo*.

It has been shown that the diatom can incorporate inorganic elements other than silicon into its frustule through active processes. In particular, our group has shown that the siliceous component of the frustule can be replaced by titanium dioxide (TiO_2_) using titanium(IV) bis(ammonium lactato)-dihydroxide (TiBALDH) as a precursor^13,14^ and that thiol moieties can be incorporated into the silica backbone via an *in vivo* modification approach. Furthermore, other studies have shown that due to structural similarity, the silica diatom can be modified through the incorporation of germanium,^15^ aluminum,^16^ cadmium,^17^ zinc,^18^ and iron,^19^ and this indicates that the diatom is a versatile organism able to change its key metabolic pathways to adapt to its surrounding environment.

Previous studies employing diatom templating approaches have focused on developing nanotopographically functionalized microparticles for the delivery of poorly soluble drugs, such as indomethacin, prednisone, mesalamine,^20^ and levofloxacin^21^ by effectively increasing the loading efficiency and percentage of drug released in the application. Furthermore, combining both small molecule or antibody attachment and drug loading^22,23^ on the surface of the diatom will allow for targeted delivery to a specific location while preventing any off-target delivery side effects. This work demonstrated that diatom is a promising template with the possibility of further improvement by tailoring the surface functionalities to optimize their application. It generates particular interest in developing biomimetic microparticles as synthetic cell systems to direct cellular activation and differential function.

**Figure 1:**
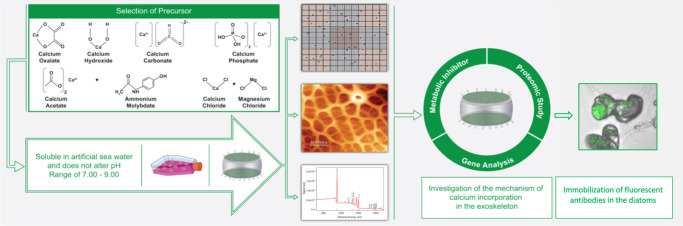
Schematic representation of the experimental design and procedure. Various calcium precursors were examined that play significant roles in the biomineralization process. The two main criteria that need to be fulfilled are that the precursor must be soluble in artificial seawater and should not increase the pH of the seawater. Once identified, the diatom cultures were fed the precursor, followed by monitoring of the cell density and characterization of the morphology and amount of calcium deposition. To characterize the mechanism underlying this phenomenon, an inhibitor study was performed to inhibit the respiratory processes that are responsible for silica incorporation and calcium, along with proteomic analysis to monitor protein regulation and gene analysis using RT-qPCR to determine the gene(s) responsible for calcium deposition. Diatoms were functionalized with polydopamine to allow the immobilization of fluorescent antibodies of CD3 and CD28 onto the surface of diatoms as a proof-of-concept for a delivery system.

In nature, *Thalassiosira weissflogii (T. weissflogii)* has a cylindrical morphology and complex 3D architecture, attracting considerable interest in biomedical applications as a platform with a high surface area.^24^ Fabricating this three-dimensional structure requires a complex synthesis that is still not fully understood; the synthetic reproduction of its features is impossible in the laboratory or sustainable as an industrial process. Here, we describe the development of a biologically inspired material using an active biochemical calcium substitution process in the *T. weissflogii* diatom (Figure 1). We demonstrated that *T. weissflogii* cultures in a calcium-rich environment resulted in calcium deposition within the diatom frustule, which retained their mimetic 3D nanoarchitecture comprising pores and protrusions ranging from 5 to 200 μm. We further investigated the mechanism of calcium deposition and showed that calcium-modified *T. weissflogii* diatoms were biodegradable. It can provide a site-specific binding capacity through surface functionalization, for example, immobilized with antibodies as present stimulatory and costimulatory T-cell activation moieties, which can be used to induce T-cell activation to rapidly establish expanded T-cell populations *in vitro* in future studies.

## Results

### Selection of Calcium Precursor in *T. weissflogii*

**Figure 2:**
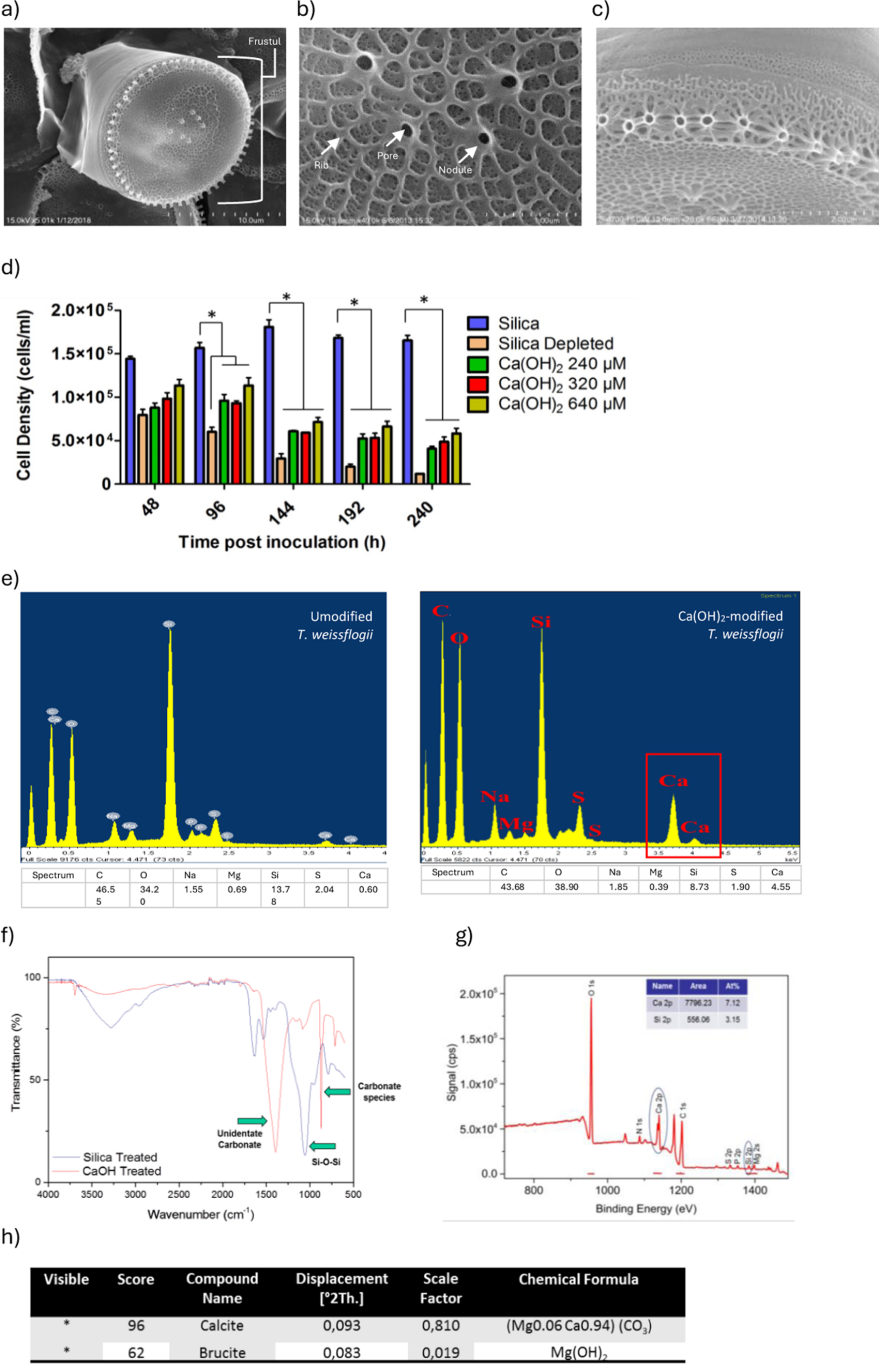
Modification of *T. weissflogii* with a calcium precursor. (a) Representative images showing the whole structure of a single frustule after the cleaning procedure. (b,c) Complex silica walls showing hierarchical patterns of pores of different sizes and shapes. (d) The growth profile of *T. weissflogii* grown in the presence of Na_2_SiO_3_ or Ca(OH)_2_ was added at 48 h intervals and revealed a significant difference at 144 h. (e) SEM-EDX confirmed a higher carbonate species in the 640 μM Ca(OH)_2_ treated diatoms compared to unmodified diatoms. (f) FTIR showed the carbonate species in the Ca(OH)_2_ treated sample at the peak of 900 cm^–1^. (g) XPS spectra of *T. weissflogii* with Ca(OH)_2_ showed the presence of a Ca peak with higher peak counts (au). (h) Chemically distinguishable footprints of the modified diatoms indicate that Ca^2+^ was deposited as CaCO_3_. *n* = 3, *P < 0.05, significant differences between the Na_2_SiO_3_ or Ca(OH)_2_ treated *T. weissflogii* with two-way analysis of variance (ANOVA) followed by Bonferroni *post hoc* analysis for (d). Data are means ± SEM. Scale bars 10, 1, and 2 μm for (a), (b), and (c), respectively.

An outline of our experimental design is shown in Figure 1. *T. weissflogii* was chosen as the model diatom for the present study since its genome was established, and the distinct frustule nanotopography with the presence of free hydroxyl groups on the surface area, allowing modification with chemical components.^13,14,25^ The surface topographical analysis confirmed the gross features of native *T. weissflogii* display distinctive fultoportulae decorating both the periphery and the core of the diatom, with its 3D frustule preserved, indicating the organic removal procedure does not affect the gross features of *T. weissflogii* (Figure 2a–c).

To find a suitable candidate for the selection of calcium precursor, five different calcium compounds, including calcium oxalate, calcium hydroxide (Ca(OH)_2_), calcium carbonate, calcium phosphate, and calcium acetate, were examined as culture additives, and their effects on diatom growth were monitored. We first tested and identified the compounds that were soluble in seawater and did not affect the pH range of seawater from 7.00–9.00. We found that only Ca(OH)_2_ was readily soluble, whereas the other compounds precipitated in artificial seawater and, in some cases, adversely affected the diatom growth profile. Therefore, we decided to use Ca(OH)_2_ for the rest of our experiment. A series of Ca(OH)_2_ concentrations ranging from 240 μM to 640 μM was explored further as potential culture medium additives. The lowest concentration, 240 μM, that we observed to affect the calcium concentration in the frustule, whereas 640 μM is the maximum dose because, above that concentration, the solution precipitates in the artificial seawater.

Bulk analysis of the diatoms revealed the mean percentage (%) of calcium incorporated into their frustules. Unmodified diatoms exhibited 2.28% ± 0.01 SEM of calcium and 15.67% ± 0.17 SEM of silica. The results indicated that the higher the treatments, the greater the percentage of silica decreases (1.83% ± 0.10 S.E.M, 1.83% ± 0.14 SEM and 1.23% ± 0.09 S.E.M) and calcium increases (27.57% ± 0.11 S.E.M, 21.74% ± 0.11 SEM and 25.27% ± 0.08 S.E.M) that were observed in the 240 μM, 320 μM and 640 μM Ca(OH)_2_-modified diatoms, respectively. This result suggests increased calcium modification of the diatoms, with calcium content showing an upward trend as silica content decreased (Table S1).

The growth patterns of *T. weissflogii* are affected by light intensity and exposure, nitrogen availability,^26^ carbon dioxide,^27^ the salinity of culture media^28^ and silica depletion.^29^ To enhance the depletion of residual silica precursors from the diatom frustule, the culture medium was depleted for 24 h without any silica solution. The experiment was then continued with multiple Ca(OH)_2_ doses added to the culture. We observed that the diatom cell density in the silica-treated group increased relative to that in the silica-depleted control conditions after 48 h of culture. The decrease in cell density at 48-h intervals was due to the sampling of cultures after adding Na_2_SiO_3_ or Ca(OH)_2_. Between silica-depleted conditions, the diatoms in the presence of Ca(OH)_2_ revealed an increased trend of cell density as Ca(OH)_2_ concentration increased (240 μM, 320 μM, and 640 μM) in comparison to the silica-depleted control group (Figure 2d), suggesting that adding Ca(OH)_2_ supports the cell growth in diatoms.

### Chemical Characterization of Calcium Deposition in *T. weissflogii*

We used surface analysis of scanning electron microscopy coupled with energy dispersive X-ray (SEM-EDX) to verify the incorporation of the Ca(OH)_2_ calcium precursor into the diatom frustule. The amount of incorporated inorganic calcium increased as a function of the Ca(OH)_2_ concentration in the seawater medium; 640 μM was observed to be the maximum calcium concentration that could be added to the culture medium without precipitation. We observed a higher calcium (Ca) peak area at position 3.5 to 4 keV in the Ca(OH)_2_-modified diatoms, confirming the incorporation of calcium into the diatom frustule (Figure 2e). Fourier-transform infrared spectroscopy (FTIR) analysis indicated the presence of silica species Si–O–Si at a peak of 1000 cm^–1^ in silica-treated diatoms. In contrast, FTIR confirmed that incorporated calcium was deposited in calcium carbonate at the peak of 900 cm^–1^ (Figure 2f). X-ray photoelectron spectroscopy (XPS) spectra analysis revealed a smaller silica (Si) 2p peak area of 556.06 (a.u), observed in silica-treated diatoms. However, the XPS spectrum of the calcium-modified diatoms further confirmed the incorporation of calcium in the diatom frustule, indicated by a higher Ca 2p peak area of 7796.23 (a.u) (Figure 2g). We then confirmed that the diatom backbone was composed of calcite with a score of 96 and a chemical formula of (Mg0.06 Ca0.94)(CO_3_) (Figure 2h), the most stable formulation of calcium carbonate.

To confirm that calcium incorporation was not a surface phenomenon, we performed bulk analysis to determine the chemical composition analysis of the Ca(OH)_2_-modified diatom (Table S1). Inductively coupled plasma mass spectrometry (ICP-MS) analysis revealed that biogenic silica in the calcium-modified *T. weissflogii* diatom was reduced from 15.67 ± 0.17% to 1.23 ± 0.09% after exposure to calcium concentrations of 640 μM, and a similar increase in calcium level from 2.28 ± 0.01% to 25.27 ± 0.08% was observed under the same conditions.

### Architectural Modification of *T. weissflogii* Frustules Following Culture in Ca(OH)_2_

**Figure 3:**
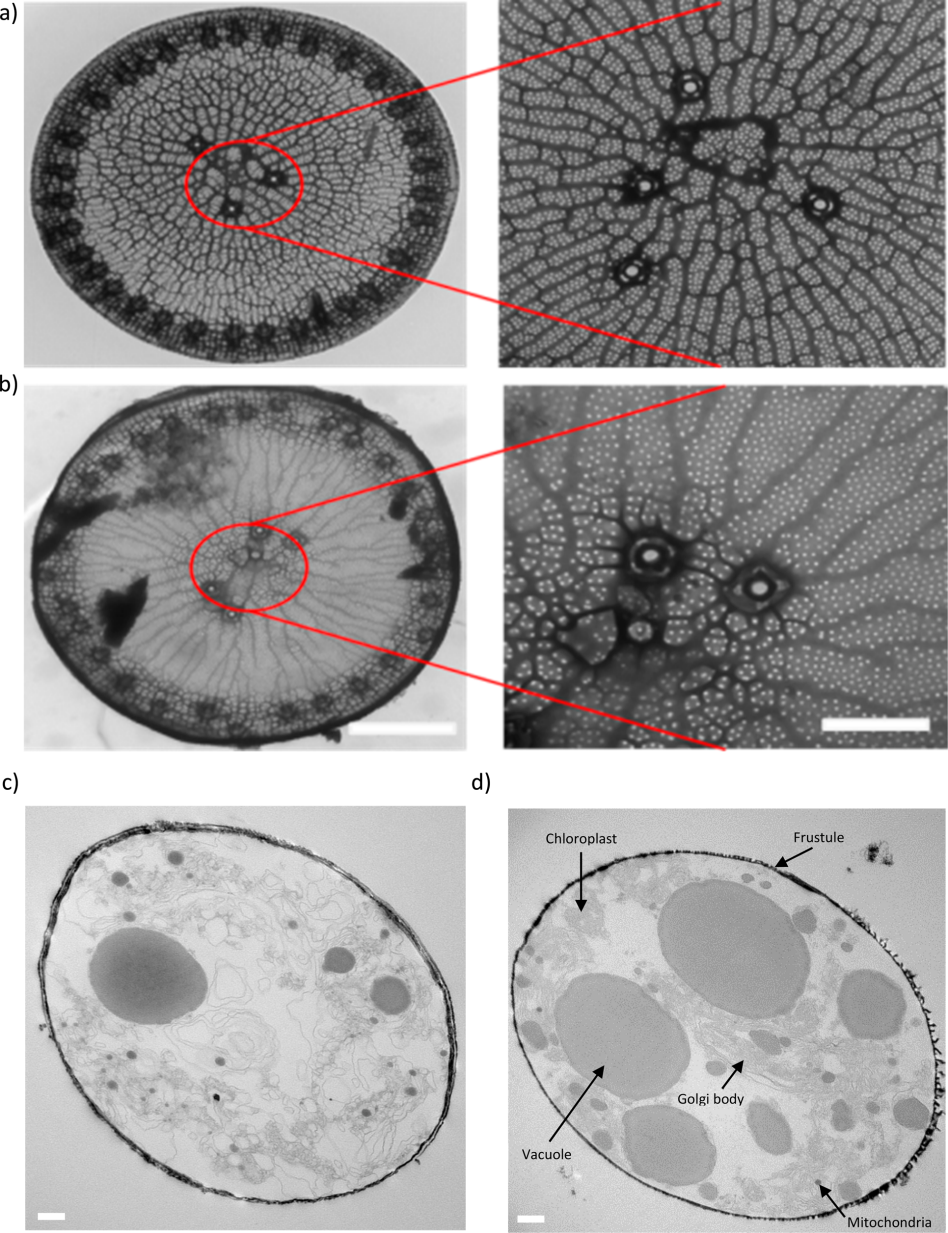

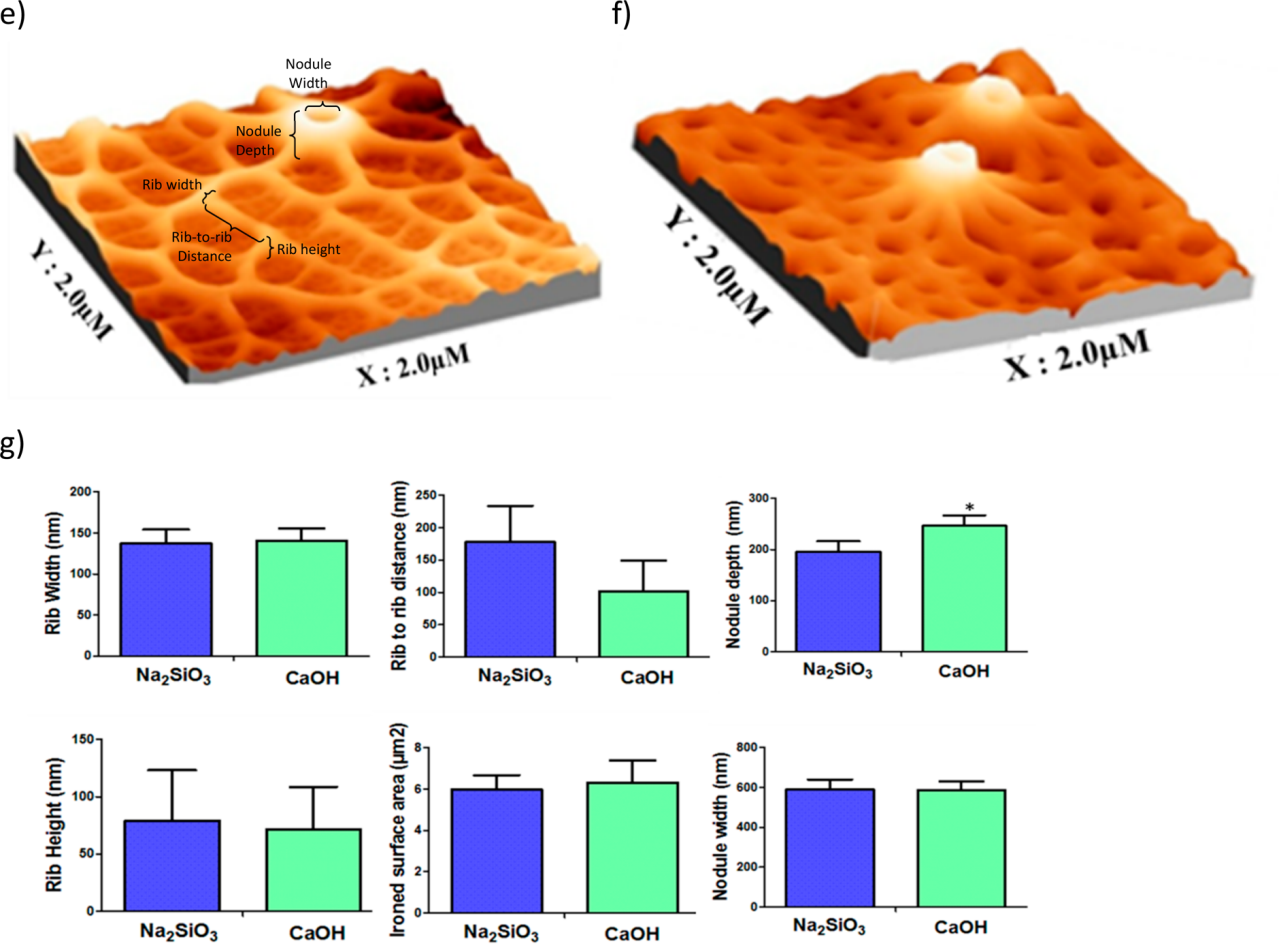
Ca(OH)_2_-modified *T. weissflogii* retains nanoscale architectural features. TEM micrographs of (a) unmodified *T. weissflogii* and (b) Ca(OH)_2_-modified *T.weissflogii* showing the unaltered nanostructure of the fultoportulae feature in the center and peripheral region. (c) TEM images of the cross-section of unmodified and (d) Ca(OH)_2_-modified diatoms indicated that the average thickness of the frustule was not significantly changed at 192 h postinoculation following multiple additions of Na_2_SiO_3_ or Ca(OH)_2_ at 0, 48, 96, and 144 h postinoculation. A typical frustule contains vacuoles, mitochondria, and Golgi bodies. Surface topography of the valve surface of (e) unmodified *T. weissflogii* and (f) Ca(OH)_2_-modified *T. weissflogii* using AFM, illustrating that the characteristic features of the gross morphology were retained. (g) Quantification data from AFM images revealed rib width, rib-to-rib distance, nodule depth, rib height, ironed surface area, and nodule width. *n* = 3 for (a,b) and (c,d), *n* = 6 for (e,g), *P < 0.05, significant differences between the Na_2_SiO_3_ or Ca(OH)_2_ treated *T. weissflogii* with *t* test for (g). Data are means ± SEM. Scale bars are 10 μm for (a,b) and 500 nm for (c,d), respectively.

We used ultraresolution microscopy to reveal the nanoarchitecture of *T. weissflogii*. Transmission electron microscopy (TEM) analysis showed that the nanostructure of the Ca(OH)_2_-modified diatoms was unaltered, similar to native *T. weissflogii* that has unique fultoportulae feature in the center and peripheral region, indicating that the Ca(OH)_2_-modified diatoms retained their complex nanoscale architecture (Figure 3a-b). The TEM cross-sectional image revealed that the average thickness of the frustule was not significantly modified (Figure 3c–d), Ca(OH)_2_-modified diatom averaging 0.12 ± 0.05 μm with an average diameter of 8.0 ± 3.0 μm with the biogenic silica diatom had an average thickness of 0.09 ± 0.06 μm with a diameter of 7.0 ± 2.5 μm. Further, atomic force microscopy (AFM) analysis confirmed no significant effects of chemical transformation with Ca(OH)_2_ on the diatom gross feature indicated by ribs radiating from the center to the peripheral region and the valve face decorated with pores at the nanometer scale (Figure 3e–f). There was a slight decrease in the rib-to-rib distance, but it was not significant. No significant differences in rib width, rib height, iron surface area, or nodule width were observable in the Ca(OH)_2_-modified diatoms, similar to the control condition. However, AFM analysis revealed a significant increase in nodule depth in the Ca(OH)_2_-modified diatoms compared to that in the Na_2_SiO_3_-treated diatoms (Figure 3g). These findings suggest that chemical modification with Ca(OH)_2_ preserves the nanoarchitecture of *T. weissflogii* with a minimal effect on nodule depth and rib-to-rib distance.

### Investigation of the Mechanism of Calcium Modification

To investigate the biosynthetic processes involved in calcium incorporation into the diatom and the mechanistic pathway involved in this phenomenon, we used an indirect method to determine whether calcium uptake derived from a passive diffusion of surrounding calcium from the seawater or whether it resulted from an active metabolic activity regulated by a calcium-ion protein transporter. To address this, we used two metabolic inhibitors, sodium azide and iodoacetamide, which are known to arrest the uptake of ion species and cell division by inhibiting aerobic respiratory processes.^30^

**Figure 4:**
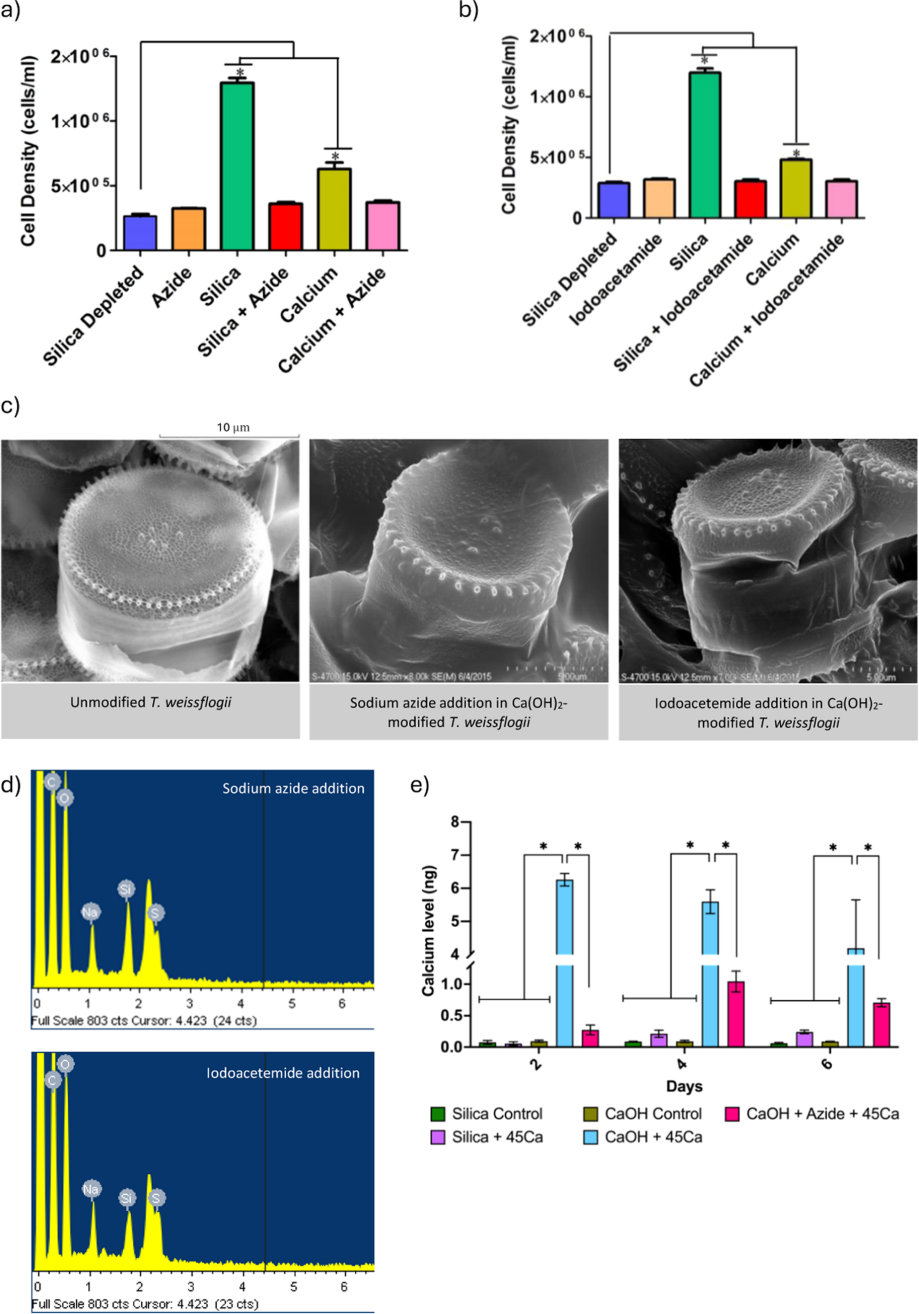

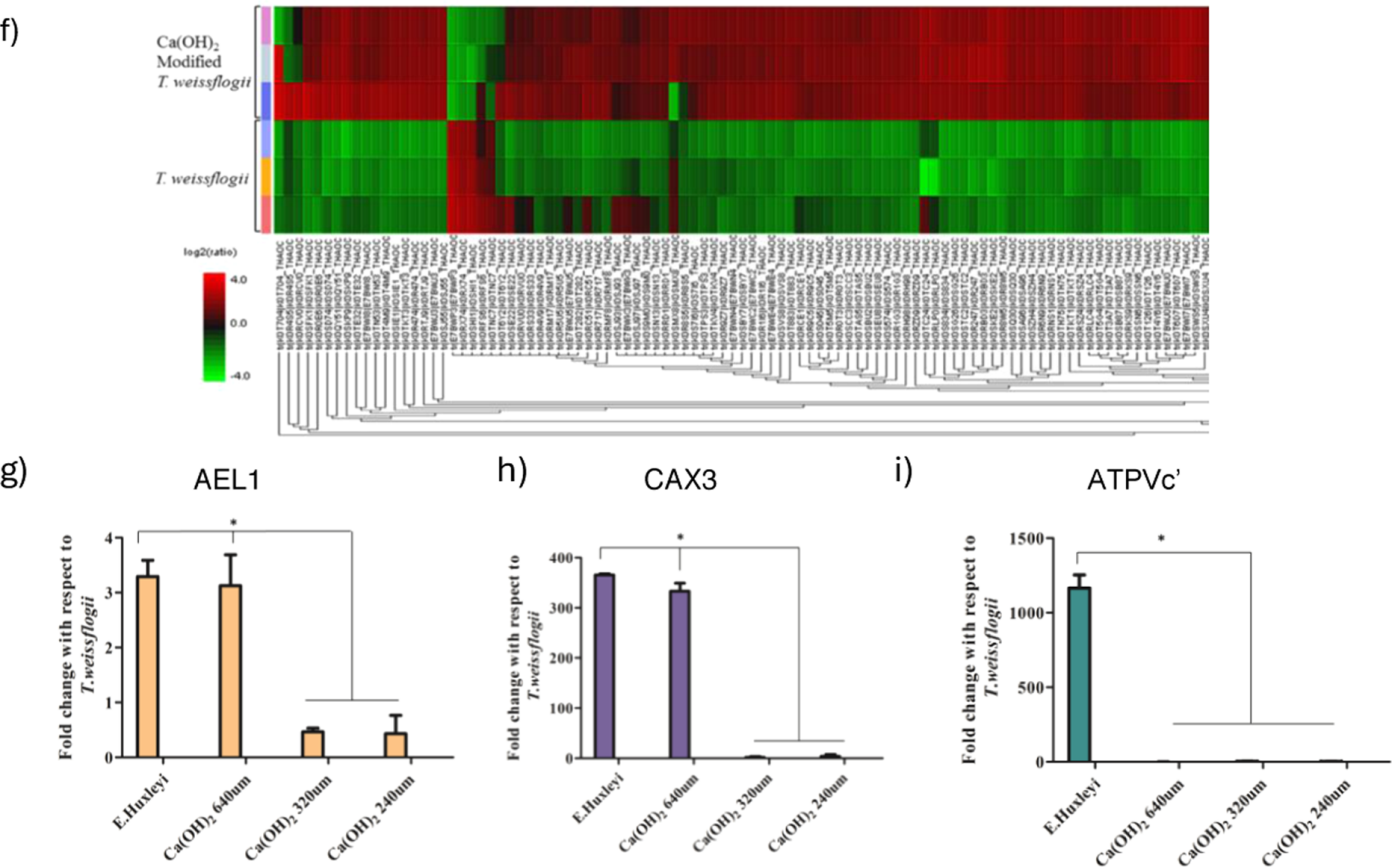
Mechanism of uptake of the Ca(OH)_2_-modified *T. weissflogii.* Two respiratory inhibitors were used to determine whether calcium uptake occurred due to active uptake or from surface phenomena. (a) Sodium azide and (b) iodoacetamide were shown to block both silica and calcium uptake from the surrounding environment into the cell membrane. (c) SEM analysis indicated no change in the surface microstructure of the Ca(OH)_2_-modified *T. weissflogii* in the presence of sodium azide or iodoacetamide. (d) SEM-EDX of Ca(OH)_2_-modified *T. weissflogii* analysis indicated the absence of a Ca^2+^ peak and an increased sulfur peak following the addition of sodium azide or iodoacetamide. (e) Radioactive uptake rate of calcium deposition in elevated treatment for six consecutive days indicated by LCS analysis. (f) Proteomic analysis showed differentially expressed proteins in Ca(OH)_2_-modified diatoms as shown in the heatmap of log2-transformed abundance generated by Peak Studio, with red for high expression and green for low expression. Gene expression analysis indicated upregulation of (g) CAX3 and (h) AEL1, and downregulation of (i) ATPVc′ was monitored to confirm the involvement of these genes in calcium deposition in diatoms. *n* = 3, *P < 0.05, significant differences between the groups with two-way ANOVA followed by Bonferroni *posthoc* analysis for (a,b), (e), and (g–i). Data are means ± SEM. Scale bar 10 and 5 μm for (c).

After culturing *T. weissflogii* in the absence or presence of Ca(OH)_2_, as well as either of the two inhibitors (10 mM concentration for each), we observed a significant reduction in cell density when the diatoms were cultured in the presence of an inhibitor, as compared to the control condition (Figure 4a–b). SEM analysis indicated no change in the surface microstructure of the calcium-modified *T. weissflogii*, in which the fultoportulae feature was preserved in the core and periphery (Figure 4c). Energy dispersive X-ray spectroscopy (EDX) analysis revealed the absence of a calcium peak and an increase of sulfur peak, which further confirmed that Ca(OH)_2_-modified diatoms cultured in 640 μM Ca(OH)_2_ with the presence of sodium azide did not incorporate calcium into the frustule (Figure 4d), indicating that incorporation was an active metabolic process and not merely due to surface absorption.

A ^45^CaCl_2_ radioactive tracer was used at 1 μCi/ml to measure the rate of calcium incorporation. Diatom cultures were inoculated in sterile deionized seawater without adding silica solution for 2 days to deplete the silica ions on the diatom. The amount of radioactive Ca^2+^ in the cells was subsequently measured by liquid scintillation counting (LSC) following washing with 20 mM MgCl_2_. Culture medium controls with unmodified seawater and the presence of a metabolic inhibitor further indicated that calcium incorporation is an active process. The Ca^2+^ level after 2 days of culture with Ca(OH)_2_ at increasing concentrations resulted in a maximum of 6.26 ± 0.33 ng of Ca^2+^ in the diatom frustule, which subsequently decreased to 5.60 ± 0.62 ng of Ca^2+^ and 4.19 ± 2.52 ng of Ca^2+^ in the diatom valve after 4 days and 6 days, respectively. In diatom cultures that were cultured in the presence of sodium azide, the Ca^2+^ level after 2 days was 0.27 ng of Ca^2+^ in the valve, and this increased to 1.04 ng of Ca^2+^ in the valve following 4 days of culture and decreased to 0.71 ng of Ca^2+^ in the valve following 6 days of culture (Figure 4e).

### Proteomic and Molecular Analysis of Calcium Uptake in *T. weissflogii*

To further study the mechanism of calcium uptake in *T. weissflogii*, proteins were extracted from three biological replicates on consecutive days and subjected to liquid-coupled tandem mass spectrometry (LC-MS/MS) analysis. Peak Studio 7 software was used to search the complete *T. weissflogii* proteome downloaded from the UniProtKB and Swiss-Prot databases. We excluded proteins identified by a single peptide and identified a total of 200 proteins in the diatom. The false discovery rate of peptide matches above a determined threshold was set at 0.58%. Heatmap showed the relative expression (log_2_ transformed) of proteins in the native and Ca(OH)_2_-modified diatoms (Figure 4f). Based on the genealogy classification, the primary proteins identified were involved in translation, metabolic processes, and photosynthesis (Table S2). In addition, approximately 30% of the proteins identified had a further annotated function assigned to them. Proteomic analysis identified a carbon-concentrating mechanism (CCM) as a potential regulator of calcium deposition after Ca(OH)_2_ treatment in *T. weissflogii* as this is a process that has been shown to act as the principal mechanism of action for calcium deposition in the single-celled phytoplankton *Emiliania huxleyi (E. huxleyi)*.^31^

To assess the role of CCM in *T. weissflogii* Ca^2+^ deposition, the expression of the three genes involved in the CCM of interest^32^ was evaluated relative to that in *E. huxleyi*. Gene expression analysis by reverse transcription-quantitative polymerase chain reaction (RT-qPCR) showed upregulation of a putative bicarbonate transporter belonging to the solute carrier four family (AEL1) and a putative calcium/proton exchanger (CAX3) in the 640 μm Ca(OH)_2_-modified *T. weissflogii* similar to *Emiliania huxleyi* (Figure 4g–h). In contrast, the expression of a putative vacuolar type 2 ATPase (ATPVc/c’) was downregulated in all conditions of Ca(OH)_2_-modified *T. weissflogii* (Figure 4i). As calcification influences the ion flux and energy demand of the cell, the transcription of genes involved in the process that supports calcification, such as increased ATP requirement, may also exhibit transcriptional regulation in addition to those directly involved in calcification.

### Immobilization of Fluorescent Antibodies on the Surface of Diatoms

**Figure 5:**
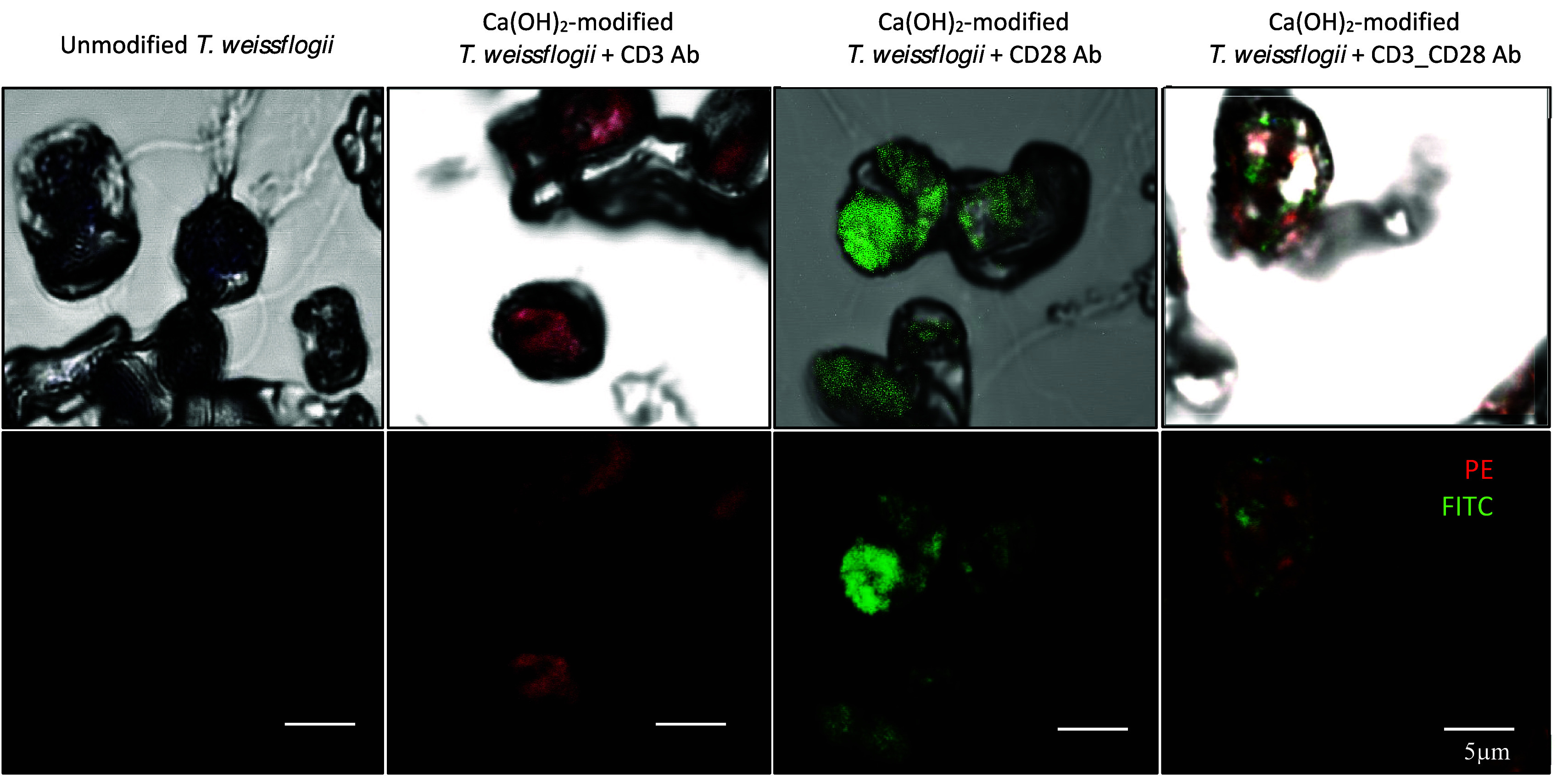
Proof-of-concept of Ca(OH)_2_-modified diatoms through antibody binding on the surface of diatoms. The fluorescence microscopy images revealed immobilization of PE-labeled CD3 (red) and FITC-labeled CD28 (green) antibodies (Ab) on the surface of Ca(OH)_2_-modified diatoms following surface functionalization with a polydopamine coating. Scale bar 5 μm.

To immobilize antibodies on the diatoms, we first functionalized the coating on the diatom surface with polydopamine to allow free amine groups for the binding of phycoerythrin (PE)-labeled cluster of differentiation (CD)3 and fluorescein isothiocyanate (FITC)-labeled CD28 antibodies on the diatoms. We demonstrated a slow weight loss of Ca(OH)_2_-modified diatoms coated with polydopamine for up to 56 days in comparison to unmodified diatoms, indicating a gradual degradation profile of polydopamine-coated modified diatoms that is suitable for the delivery system over a long time (Figure S1). Compared to unmodified *T. weissflogii*; fluorescent microscopy revealed the localized immobilization of the CD3 and CD28 antibodies on the surface of Ca(OH)_2_-modified *T. weissflogii* (Figure 5). This result suggests the proof-of-concept of Ca(OH)_2_-modified *T. weissflogii* in advancing the delivery system through antibody binding on the surface of the diatoms.

## Discussions

Our preliminary findings revealed that calcium can intentionally accumulate within the diatom T. weissflogii, contrary to conventional wisdom. Despite this process occurring within the organism, our results indicate that the three-dimensional structure of the frustule remains undamaged. This study demonstrates that calcium uptake occurs through an active mechanism rather than simply adhering to the surface.

Five calcium compounds were selected for this study: calcium oxalate, calcium hydroxide, calcium carbonate, calcium phosphate, and calcium acetate. Calcium oxalate was investigated due to previous findings of a polypeptide (silacidin) with a structure similar to that of the diatom that exists in plant biomineralization processes involving amorphous inorganic phases of silica and calcium oxalate.^33,34^ This structural composition holds the promise that following the uptake of the precursor, there is potential for polypeptide-directed calcium oxalate precipitation in the diatom frustule. Diatoms commonly use chitin to strengthen their cell walls or skeletons.^35^ Chitin is the most abundant polymer in the ocean and consists of a chemical group of C = O, O–H, and N–H bonds with an affinity for silicate ions. Importantly, chitin has an affinity for calcium and hydroxyl ions of the corresponding calcium phase, which are available from calcium hydroxide precursors.

Calcium carbonate was also tested because multiple biomineralization processes of biocomposites of silica-chitin-aragonite (in the form of calcium carbonate) can coexist together (silification and calcification).^36,37^ Calcium phosphate was tested, as polypeptides (silacidin) with structures similar to the diatom are involved in different biomineralization processes contributing to the amorphous inorganic phases of calcium phosphate.^38^ Diatoms can also precipitate calcium phosphate if the identical polypeptides are present. Finally, calcium acetate with ammonium molybdate was tested, as the diatom possesses a peptide sequence shown to crystallize (precipitate) calcium molybdate *in vitro*.^39^ Among these compounds, only Ca(OH)_2_ was soluble in the seawater medium without affecting the pH; therefore, we conducted a range of Ca(OH)_2_ concentrations from 240 μM to 640 μM throughout the study.

There are multifactorial contributors to the cell growth in diatoms. We introduced multiple doses of Ca(OH)_2_ following silica depletion at 24 h. It has been demonstrated that control overgrowth can be achieved through synchronization, whereby the cells are starved from silica solution, causing the cell wall to arrest.^29^ The reintroduction of silica leads to a surge in silica uptake and almost immediate division of cells.^40^ Compared to the silica-depleted control conditions, Ca(OH)_2_ treatment at a maximum concentration of 640 μM confirmed that the presence of a calcium precursor did not adversely affect the growth pattern of *T. weissflogii*. This knowledge may allow the exploitation of certain aspects of the cell growth process to alter the silica content of diatoms, alter the morphology of diatoms, and to some extent, modify the elemental composition of the frustule.

Chemically, extensive surface analyses revealed higher Ca peak counts and carbonate species following Ca(OH)_2_ treatment in *T. weissflogii* culture, confirming calcium deposition in these diatoms. It indicates that Ca^2+^ became incorporated into the silica backbone of the diatom frustule as calcite, a stable form of calcium carbonate abundant in marine microorganisms that undergo calcification, such as sponges,^41^ foraminifera^42^ and cocolithophores.^43^ Although significant Ca^2+^ deposition was observed following diatom culture in a calcium-rich environment, no significant modulation of diatom morphology was observed; however, complete substitution of the silicon dioxide (SiO_2_) valve was not achieved. The associated decrease observed in the growth rate of the Ca(OH)_2_-modified diatom, attributed to exposure to Ca^2+^, had a slight negative impact on the essential cellular process necessary for cell division due to the lack of substrate for calcification.

Morphologically, the nanoarchitecture of Ca(OH)_2_-modified *T. weissflogii is* still preserved, with its unique three-dimensional (3D) frustule feature, ribs projecting from the core to the periphery, and pores decorating its valve. The frustule thickness remained similar to that of the unmodified *T. weissflogii*. Although we observed minimal alterations in nodule width and rib-to-rib distance, the rib’s gross features of height and width, and the surface area were unaltered in Ca(OH)_2_-modified *T. weissflogii*. Our results are in line with our previous studies on thiol-modified *T. weissflogii, which* showed fultoportulae features in diatom; however, the minimal alterations in the pore parameters due to changes in the siloxane backbone of the frustule.^13,14^ Similarly, CaCl_2_-doped biosilica demonstrated an unaltered nanostructure of the diatom *T. weissflogii* with pores remaining empty.^44^

We utilized ^45^CaCl_2_ radiotracer to confirm calcium uptake and deposition in the diatoms. Here, the glycine-high calcium medium mimicked the physiological environment to prevent further calcium uptake, which is in line with the previous study.^45^ Glycine is a nonessential amino acid that causes an influx of chloride, thereby impeding the opening of voltage-gated Ca^2+^ channels^46^ to ultimately block the increase in intracellular free Ca^2+^. Our finding indicated Ca(OH)_2_-treated diatoms exhibited a higher amount of Ca^2+^ compared to Ca(OH)_2_-modified diatoms in the presence of inhibitor (sodium azide) or silica-depleted diatoms as control, confirming the calcium deposition in the Ca(OH)_2_-treated diatoms.

Critically, remodeling the diatom silica chemistry was associated with significant changes in crucial calcification-associated metabolic pathways. Carbon acquisition processes, carbonic anhydrase (CA),^47^ stress, degradation, and signaling proteins were upregulated in diatoms exposed to Ca(OH)_2_ treatment, corroborating previous evidence that CCM during C_4_ photosynthesis is vital in *T. weissflogii* metabolism.^48^ Furthermore, metabolic inhibition abolished calcium and silica deposition, a photosynthesis-dependent process. This result suggests that Ca^2+^ calcification most probably enters the cell down its electrochemical gradient via a Ca^2+^ permeable channel. Loading the endomembrane compartment with Ca^2+^ requires either AP-dependent pumping or ion exchange using the electrochemical gradient of another ion species. The upregulation of CAX3 supports this as a potential role in calcification for Ca^2+^ loading via the H^+^ gradient.

Previously, it has been shown in unicellular algae that the calcification process results in proton generation, which drives the excessive generation of CO_2_ and deposition of calcium carbonate from bicarbonate.^49^ Interestingly, ribulose bisphosphate carboxylase oxygenase (RuBisCO), the enzyme responsible for CO_2_ assimilation in microalgae and cyanobacteria, was upregulated in diatoms exposed to calcium-rich environments (Table S2).

**Figure 6:**
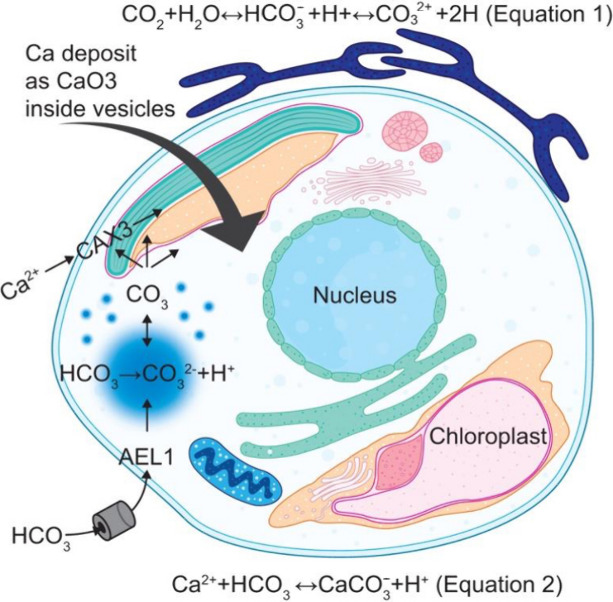
Schematic representation of the mechanism of calcium uptake in *T. weissflogii*. Calcium carbonate (CaCO_3_) precipitation requires the production of carbonates (CO_3_^2–^) from bicarbonate (HCO_3_^–^) and results in the net production of H^+^. Calcium uptake into cells involves Ca^2+^ transporters; Ca^2+^ ions are concentrated into a compartment distinct from the vesicle–reticular body system.

Several genes encoding proteins with putative roles in carbonate, Ca^2+^, and H^+^ transport (AEL1, CAX3, and ATPVc’/c) were investigated to evaluate the mechanism involved. Diatoms possess multiple homologues of AEL1^50^ that have been shown to play a role in calcification by stimulating HCO_3_ uptake and photosynthesis. The upregulation of this gene, together with CAX3, which is responsible for Ca^+^/H+ exchange, further proves that calcium carbonate deposition utilizes HCO_3_ as the primary substrate for calcification (Figure 6). A previous study demonstrated that calcification in *E. huxleyi* is primarily driven by HCO_3_.^32^

The immobilization of antibodies onto the diatoms was achieved through a reaction between the catechol and primary amine groups of the polydopamine structure and the amine and thiol groups of the antibody.^51^*In situ* polydopamine-based artificial coating on the surface of diatoms has previously been demonstrated without affecting diatoms growth kinetics.^52^ Here, we successfully immobilized CD3 and CD28 antibodies on polydopamine-coated diatoms, which indicated the feasibility of utilizing Ca(OH)_2_-modified *T. weissflogii* for antibody-specific binding on the surface of diatoms, potentially employed for stimulatory T-cell activation and population in immunotherapy. Previous studies employed anti-CD3 and anti-CD28-coated beads to induce human T-cell expansion and activation *in vitro*.^53^

## Conclusions

Through the chemical substitution of calcium (Ca(OH)_2_) within the diatom frustule, we successfully modified the structure of the diatom while preserving its intricate nanoscale architecture. This process involves calcium uptake via a ’carbon-concentrating mechanism’ and does not involve any surface-level changes. Our findings yield promising results for developing next-generation biomaterials inspired by natural processes. These materials, which are biodegradable and utilize natural resources, have the potential to bring about significant benefits in the field of material science.

## Methods

### Cultivation of *T. weissflogii*

Axenic *T. weissflogii* cultures were grown in artificial seawater (ASW), prepared according to Berges et al.,^54^ and enriched with Guilard’s f/2 marine enrichment medium without silicates (Sigma-Aldrich) according to the manufacturer’s recommendations. Cultures were silica-depleted according to the procedure detailed by Hildebrand et al.^55^ for a minimum of 24 h before inoculation with the precursor. Following silica depletion, the cultures were inoculated at 1 × 10^4^ cells/ml or 5 × 10^4^ cells/ml in a final volume of 200 mL. Cultures were grown in polystyrene tissue culture flasks. Sodium metasilicate nonhydrate (Na_2_SiO_3_) or Ca(OH)_2_ in the form of calcium oxide (CaO) was added to the cultures at a final concentration of 640 μM. The cultures were grown in a 14 h:10 h light:dark cycle at a light intensity of 3000 lx and a temperature range of 16–22 °C. Multiple-dose treatment cultures received further addition of Na_2_SiO_3_ or Ca(OH)_2_ at final concentrations of 640 μM at 48, 96, and 144 h. Cultures were collected 192 h postinoculation, and the diatoms were cleaned. Cell density was monitored using a hemocytometer.

### Materials and Reagents

Sodium metasilicate nonhydrate, 7-fluorobenzo-2-oxa-1,3-diazole-4-sulfonic acid ammonium (SBDF), dimethyl sulfoxide, methanol, buffer reagents, trypsin, artificial seawater (ASW) reagents, and calcium-45 radionuclide were purchased from Sigma-Aldrich (Ireland). Lysosensor Yellow/blue DND-160 (PDMPO) was purchased from Invitrogen (France). Random primers, reverse transcriptase, 5× Reaction Buffer, 25 mM MgCl_2_, dNTP mix, and recombinant RNasin ribonuclease were purchased from Bioline (Dublin, Ireland). Anti-CD3 (clone PC3/188A), anti-CD28 (clone CD28.2), anti-CD69 (clone FN50) and anti-CD4 (clone H-370) were purchased from Abcam (Ireland).

### Preparation of Cleaned Frustules for Characterization

Organic matter was removed from the diatoms by successive washing with heated deionized water (60 °C), deionized water, and methanol. Diatoms were suspended in heated deionized water for 20 min, followed by centrifugation at 2,500 g for 20 min. This process was repeated three times. Three washes in deionized water were performed. The final cleaning step involved a minimum of three washes with methanol until the pellet appeared white. The cleaned frustules were subsequently examined by SEM–EDX, TEM–EDX, XPS, Inductively Coupled Plasma Optical Emission spectroscopy (ICP-OES), and AFM analyses.

### Characterization of Frustules by Scanning Electron Microscopy with Energy Dispersive X-ray Analysis (SEM–EDX)

Cleaned frustules suspended in methanol were air-dried on a carbon stub and subsequently coated with gold. SEM–EDX analysis was performed using a Hitachi S-4700 SEM with INCA software (Oxford Instruments). Frustules were analyzed on valve faces that were visible.

### Characterization of Frustules by Transmission Electron Microscopy with Energy Dispersive X-ray Analysis (TEM–EDX)

The TEM-EDX analysis was performed with a JEOL 2100F electron microscope operated at 200 kV using a field-emission electron source equipped with a Gatan Ultrascan Camera. Cleaned frustules suspended in methanol were allowed to air-dry on a copper grid. The identity of each element was confirmed using an EDAX detector and the Genesis software. The characterization of the pore parameters and pore distribution was performed using ImageJ software on TEM images collected via a Hitachi H-7500 TEM with AMT image capture software. *t* tests were performed to compare differences between the treatment groups.

### Characterization of Frustules by X-ray Photoelectron Spectroscopy (XPS)

The cleaned frustules were then dried at 60 °C for 48 h. The samples were dusted onto a double-sided adhesive and analyzed with a Kratos AXIS-165 spectrometer using monochromatic Al Kα radiation. The XPS analyses were calibrated with respect to the carbon 1s excitation (284.8 eV).

### Characterization of Frustules by Inductively Coupled Plasma-Optical Emission Spectrometry (ICP-OES)

Diatoms, CRM 414, and a reagent blank were refluxed on a hot plate in closed Teflon beakers using analytical-grade nitric acid and hydrofluoric acid. After dissolution, the mineral acids were evaporated to dryness and dissolved in 5% HNO_3_ before analysis. Ca^2+^ concentration was determined using inductively coupled plasma optical emission spectroscopy. The mean values of three repeated analyses are reported, and the analytical uncertainty was expressed as the standard deviation of these three analyses from the mean. The accuracy was determined by tandem digestion and analysis of the plankton-certified reference material “CRM 414”. The recovery of Ca^2+^ from CRM 414 was 103.8% of the indicative value.^56^

### Flow Injection Analysis (FIA) of Biogenic Silicon

Samples were digested in a water bath for 2 h at 80 °C using 0.5 M sodium hydroxide.^57^ The Si concentrations were determined using a flow injection auto analyzer. The mean values of the three repeat analyses were reported as a percentage (%), and the analytical uncertainty was expressed as the standard deviation of these three analyses from the mean. Precision and accuracy were estimated using repeat analysis.

### Characterization of Frustules by Atomic Force Microscopy (AFM)

The AFM measurements were performed under ambient conditions in intermittent contact mode using a NanoWizard-II AFM coupled with an inverted optical microscope. Silicon cantilevers (spring constant, kB2.8 N m_1, and resonance frequency, fB75 kHz) with a high aspect ratio (1:10 aspect ratio, tip radius 0.3 nm), high density, and diamond-like carbon tips were used (MSS-FMR-13, Nanotools, Germany). AFM images were analyzed using WSxM Software29.

### Metabolic Inhibitory Study

*T. weissflogii* samples were prepared for culture as detailed in the cultivation method. The concentration of inhibitors was inspired by our previous study.^58^ Sodium azide (10 mM) and iodoacetemide (10 mM) were incubated in the culture for 4 days to prevent metabolic uptake throughout the experiment. The cells were counted using a hemocytometer, and SEM-EDX was used to further evaluate the diatom structure.

### ^45^Ca Radioisotope Study

Radioisotope uptake rates were performed using Hitachi liquid scintillation. The radioisotope used in this study was ^45^Ca (as [^45^CaCl_2_], 5 μCi) obtained from PerkinElmer. The diatoms were starved in 100% ASW without adding the precursor and incubated with the radioisotope the following day. At the end of the labeling period, cultures were immersed for 20 s in a beaker containing 600 mL ASW and rinsed five times with 5 mL ice-cold glycine-rich calcium medium (50 mM CaCl_2_, 950 mM glycine, pH adjusted to 8.2) to prevent further uptake and reduce isotopic dilution from ^45^Ca adsorbed on the external surface of the diatom.^45^

### Proteomic Analysis by Liquid-Coupled Tandem Mass Spectrometry (LC-MS/MS)

Proteins were extracted from diatoms using trichloroacetic acid (TCA) protein precipitation. To extract proteins, samples were mixed with one volume of TCA into four volumes of protein samples. The samples were then incubated for 10 min at 4 °C and centrifuged in the microcentrifuge tubes at 14,000 rpm for 5 min. The supernatant was discarded, leaving only the intact pellets. The pellet was then washed with 200 μL of cold acetone and centrifuged at 14,000 rpm for 5 min. After that, the pellet was rewashed with acetone and dried in the tube in a 95 °C heat block for 5–10 min to remove acetone. The pellet was resuspended in 50 μL of 6 M urea in ammonium bicarbonate. The sample was then digested in trypsin solution with a trypsin: protein working ratio of 1:50.

The samples were run on a Thermo Scientific Q Exactive mass spectrometer connected to a Dionex Ultimate 3000 RSLCnano chromatography system. Tryptic peptides were resuspended in 0.1% (v/v) formic acid. Each sample was loaded onto a fused silica emitter (75 μm internal diameter, pulled with a laser puller (Sutter Instruments P2000), packed with Reprocil Pur C18 (1.9 μm) reverse-phase medium), and separated with an increasing acetonitrile gradient over 47 min at a flow rate of 250 nL/min. The mass spectrometer was operated in positive-ion mode, with a capillary temperature of 320 °C and a potential of 2,300 V applied to the frit.^59^ All data were acquired with the mass spectrometer operating in the automatic data-dependent switching mode. A high-resolution (70,000) MS scan (300–1,600 *m*/*z*) was performed with Q Exactive to select the eight most intense ions before MS/MS analysis with higher-energy collisional dissociation. For protein identification, the raw data were searched against the *Thalassiosira* subset of the UniProt Swiss-Prot database, using the search engine PEAKS Studio 7 (Bioinformatics Solutions, Waterloo, ON, USA) for peptides. Each peptide for protein identification met specific PEAKS parameters; only peptide scores corresponding to a false discovery rate (FDR) of ≤1% were accepted from the PEAKS PTM database search.

### Gene Expression Analysis by Reverse Transcription Quantitative Polymerase Chain Reaction (RT-qPCR)

Diatom samples were homogenized in 1 mL of TRIzol reagent per 5–10 mg of the tissue sample using a TissueLyser (Qiagen). The samples were shaken at high speed in 2-ml round-bottom microcentrifuge tubes with stainless-steel beads for 15 min at room temperature. The mixture was then centrifuged at 13,300 rpm for 15 min at 4 °C. The colorless upper aqueous phase containing ribonucleic acid (RNA) was transferred to a fresh tube and mixed with 600 μL of 70% molecular-grade ethanol. The mixture was transferred to a Qiagen RNeasy Mini column and centrifuged at 10,000 rpm for 15 s, and the flowthrough was discarded. RNA bound to the column was washed with 700 μL of RW1 buffer and centrifuged at 10,000 rpm for 15 s. Next, 500 μL of RPE buffer was added to the column, followed by centrifugation at 10,000 rpm for 15 s, followed by a further addition of 500 μL of RPE buffer and centrifugation at 10,000 rpm for 2 min. RNase-free water (30 μL) was added, and the mixture was centrifuged at 10,000 rpm for 1 min to collect RNA in a new collection tube. The RNA concentration was determined with a NanoDrop spectrophotometer (Thermo Scientific) from the ratio of the absorbance at 260 and 280 nm, and the quality of the product was determined by using a Bioanalyser (Agilent, Santa Clara, CA, USA). Total RNA (100 ng/μL) was reverse-transcribed with random primers and reverse transcriptase in a 20-μL reaction mixture consisting of 5× reaction buffer, 25 mM MgCl_2_, dNTPs, and RNasin ribonuclease inhibitor, with the PTC DNA Engine System (PTC-200, Peltier Thermal Cycler, MJ Research, Watertown, MA, 28 USA). cDNA products were amplified SYBR green PCR Master Mix (Promega) and following specific *CAX3*, *AEL1*, and *ATPV’c primers*. A PCR reaction was performed in triplicate using the StepOnePlus Real-Time PCR System (Applied Biosystems)^60^ with standard thermal conditions (5 min at 95 °C for polymerase activation, followed by 40 cycles of 95 °C for 15 s and 60 °C for 30 s and one cycle of melt curve stage of 95 °C for 15 s and 60 °C for 60 s). The results were analyzed by the 2–^ΔΔCt^ method, and the results were normalized to those of *T. weissflogii* as a control. Primer’s sequence of each gene: Glyceraldehyde 3-phosphate dehydrogenase (GAPDH) forward TACTGCGATGAGCCTTGTGTG, reverse GAACTTGGGGTTGAGGGAGA; CAX3 forward CTCCTCTGCGTCTTTGCAT, reverse GTTCAGCGTGCTCTCCGAG; AEL1 forward TTCACGCTCTTCCAGTTCTC, reverse GAGGAAGGCGATGAAGAATG; ATPV’c forward ACGGGGATGATGGACTTC, reserve CTCCTCTGCGTCTTTGCAT.

### Degradation Study in Phosphate Buffer Saline (PBS)

Equal weights of diatom templates were prepared to measure their dry weight (W_d_). Degradation was evaluated using wet/dry loss (%). The samples of unmodified *T. weissflogii*, Ca(OH)_2_-modified *T. weissflogii*, and Ca(OH)_2_-modified *T. weissflogii* coated with polydopamine were removed from the PBS solution after 3, 7, 14, 28, 56, and 112 days of degradation. Their dry weights were measured as follows:
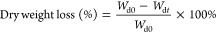
where W_d0_ and W_dt_ are the weights of the dry materials before and after degradation, respectively.

### Immobilization of Fluorescent Antibodies on the Surface of *T. weissflogii* Using Fluorescent Microscopy

The unmodified diatoms and Ca(OH)_2_-modified diatoms were first functionalized with a polydopamine coating at an optimal 1:3 ratio of diatom: polydopamine for 1 h, thus providing free amine groups to facilitate the conjugation of PE-labeled CD3 and FITC-labeled CD28 antibodies on the diatoms. This ratio and coating time provided sufficient (300 μg/mL) amine groups on the surface to allow antibody conjugation. The CD3 (0.1 μg/mg diatom) and CD28 (0.5 μg/mg diatom) antibodies were incubated in the samples of polydopamine-coated diatoms for 2 h at 37 °C. The samples were washed with phosphate buffer saline with Tween20 (PBS-T) before spreading on the glass slides and coverslip-mounted. All slides were cured in the dark while imaging with an inverted fluorescent microscope (Olympus IX81, Olympus Optical Co. Ltd.).

#### Statistical Analysis

Statistical analysis was performed using GraphPad Prism version 5.00 software. Data were compared by *t* test for quantification data from AFM images and two-way analysis of variance (ANOVA), and multiple pairwise comparisons were performed using the Bonferroni *post hoc* for cell density, gene expression analysis, percentage of calcium incorporated into diatom and degradation study. Statistical significance was set at *P* < 0.05.

## Supporting Information Available

Table S1 – Bulk analysis of the diatoms. Percentage (%) of calcium incorporated into diatom frustules. The higher the treatment, the greater the percentage of silica decreases and calcium increases. Table S2 – Proteomic analysis of the diatoms. Changes in the relative abundance of proteins in *T. weissflogii* after Ca(OH)_2_ treatment. Figure S1 – Degradation study of the diatoms. Dry weight loss rates (%) of the unmodified diatoms, Ca(OH)_2_-modified diatoms, and Ca(OH)_2_-modified diatoms coated with polydopamine at (a) pH 6 and (b) pH 5 (PDF)

## Abbreviations

CaCO_3_: calcium carbonate
Ca(OH)_2_: calcium hydroxide
CD: cluster of differentiation
PDMPO: 2-(4-pyridyl)-5-((4-(2-dimethylaminoethylaminocarbamoyl)methoxy)phenyl)oxazole
*T. weissflogii*: *Thalassiosira weissflogii*
TEMPO: tetramethylpiperidine-*N*-oxyl
TiO_2_: titanium dioxide
TiBALDH: bis(ammonium lactato)-dihydroxide
SEM: standard error of mean
FTIR: Fourier-transform infrared spectroscopy
XPS: X-ray photoelectron spectroscopy
Si: silica
Ca: calcium
Mg: magnesium
ICP-MS: inductively coupled plasma mass spectrometry
TEM: transmission electron microscopy
AFM: atomic force microscopy
EDX: energy dispersive X-ray spectroscopy
LSC: liquid scintillation counting
CaCl_2_: calcium chloride
LC-MS/MS: liquid-coupled tandem mass spectrometry
CCM: carbon-concentrating mechanism
RT-qPCR: reverse transcription-quantitative polymerase chain reaction
AEL1: putative bicarbonate transporter belonging to the solute carrier four family
CAX3: putative calcium/proton exchanger
ATPVc/c′: putative vacuolar type 2 ATPase
PE: phycoerythrin
FITC: fluorescein isothiocyanate
SiO_2_: silicon dioxide
3D: three-dimensional
CA: carbonic anhydrase
RuBisCO: ribulose bisphosphate carboxylase oxygenase
*E. huxleyi*: *Emiliania huxleyi*
ASW: artificial seawater
Na_2_SiO_3_: sodium metasilicate nonhydrate
CaO: calcium oxide
ICP-OES: inductively coupled plasma optical emission spectroscopy
TCA: trichloroacetic acid
FDR: false discovery rate
RNA: ribonucleic acid
GAPDAH: glyceraldehyde 3-phosphate dehydrogenase
PBS: phosphate buffer saline
PBS-T: phosphate buffer saline with Tween20
ANOVA: two-way analysis of variance
