# Supplementary material for: Modification of Living Diatom, Thalassiosira weissflogii, with a Calcium Precursor through a Calcium Uptake Mechanism: A Next Generation Biomaterial for Advanced Delivery Systems
Source: ACS Appl Bio Mater. 2024 May 17;7(6):4102–15. doi: 10.1021/acsabm.4c00431 (PMC11190972; doi:10.1021/acsabm.4c00431)
Supplement: Supplementary file 1 — mt4c00431_si_001.pdf [file mt4c00431_si_001.pdf]

Supplementary Information

Modification of Living Diatom, *Thalassiosira weissflogii* with Calcium Precursor through Calcium Uptake Mechanism: A Next Generation Biomaterial for Advanced Delivery System

Asrizal Abdul Rahman[1]*, Isma Liza Mohd Isa*[1,2]*, Syed A. M. Tofail* [3]*, Lukasz Bartlomiej* [4]*, Brian J. Rodriguez*[4]*, Manus J. Biggs*[1]*, Abhay Pandit*[1*]

[1]CÚRAM, SFI Research Centre for Medical Devices, University of Galway, Ireland

[2]Department of Anatomy, Faculty of Medicine, Universiti Kebangsaan Malaysia

[3]Materials and Surface Science Institute, University of Limerick, Ireland

[4]Conway Institute of Biomolecular and Biomedical Research and School of Physics, University College Dublin, Ireland

*Corresponding to abhay.pandit@universityofgalway.ie

Table S1. Bulk analysis of the diatoms. Percentage (%) of calcium incorporated into diatom frustules. The higher the treatment, the greater the percentage of silica decreases and calcium increases. Data are presented as the means ± S.E.M. (*n*=3). Two-way ANOVA followed by Bonferroni *post hoc* analysis revealed significant differences at $^*P < 0.05$.

	**Calcium (%) Mean ± sem**	**Silica (%) Mean ± sem**
T. weissflogii	2.28 ± 0.01	15.67 ± 0.17
**$Ca(OH)_2$-modified *T. weissflogii* – 240 µM**	27.57 ± 0.11 *	1.83 ± 0.10 *
**$Ca(OH)_2$-modified *T. weissflogii* – 320 µM**	21.74 ± 0.11 *	1.83 ± 0.14 *
**$Ca(OH)_2$-modified *T. weissflogii* – 640 µM**	25.27 ± 0.08 *	1.23 ± 0.09 *

Table S2. Proteomic analysis of the diatoms. Changes in the relative abundance of proteins in *T. weissflogii* after $Ca(OH)_2$ treatment.

**Regulated proteins based on two or more peptides at >95% confidence**					
**Accession**	**Annotation**	**Go Process**	**Peptides**	**Unique**	**Significance (-log*P*)**
**A0A089VPED**	Photosystem II protein D1	Electron transport	8	7	119.71
**A0A089VIM8**	ATP synthase subunit alpha, chloroplast	ATP synthesis, Hydrogen ion transport, Ion transport	16	11	119.37
**K0RIC9**	ATP synthase subunit beta	ATP synthesis	19	17	119.00
**A0A089VIJ5**	Photosystem I P700 chlorophyll a apoprotein A2	Electron transport, Photosynthesis, Transport	15	13	118.43
**R1DMT8**	Transketolase	Transferase activity	11	11	118.07
**E7BWF9**	Photosystem II CP43 reaction centre protein	Photosynthesis	12	12	117.69
**K0SJ55**	V-type proton ATPase subunit a	Hydrogen ion transport	6	6	117.54
**R1DH33**	ADP-ribosylation factor	G.T.P. binding	8	8	116.96
**B8CFG5**	Fucoxanthin chlorophyll a/c protein 4	Photosynthesis	5	1	116.89
**B8BV47**	RL7, ribosomal protein 7	Ribonucleoprotein	5	5	115.88
**B8C553**	Adenosylhomocysteinase	One-carbon metabolism	6	6	115.08
**B8LE41**	Triose or hexose phosphate / phosphate translocator	Integral component of membrane	2	2	115.08
**B8BZD7**	RS16, ribosomal protein 14 40S small ribosomal subunit	Ribonucleoprotein	7	7	114.73
**B8C0K5**	RS13, ribosomal protein 13 40S small ribosomal subunit	Ribonucleoprotein	4	4	114.37
**Accession**	**Annotation**	**Go Process**	**Peptides**	**Unique**	**Significance (-logP)**
**B8BUE1**	Vacuolar membrane proton pump, inorganic pyrophosphatase	Hydrolase	9	9	114.19
**R1E2V4**	Peptidylprolyl isomerase	Isomerase	15	15	112.70

**A0A089X953**	Cytochrome b559 subunit alpha	Electron transport	3	3	112.35
**A0A089VKD8**	Ribulase-1,5-bisphosphate carboxylase / oxygenase small subunit	Chloroplast	10	9	111.45
**A0A089X8Z4**	Cytochrome c-550	Electron transport, Photosynthesis, Transport	5	5	110.19
**K0R8W5**	Clathrin heavy chain	Clathrin coat assembly, intracellular protein transport, vesicle-mediated transport	21	21	109.11
**A0A089VKI3**	Photosystem II D2 protein	Electron transport, Photosynthesis, Transport	6	6	106.42
**A0A089VP86**	Photosystem 1 assembly protein Ycf4	Photosynthesis	5	5	106.26
**B8C415**	Oxygen-evolving enhancer protein 1	Calcium ion binding, Photosynthesis, Photosystem II stabilization	8	8	105.61
**A0A089VQE1**	Cytochrome b6	Electron transporter, Photosynthesis, Transport	5	5	104.90
**B5YNM6**	RL21, ribosomal protein 21, 60S large ribosomal subunit	Ribonucleoprotein	4	3	104.84
**K0R474**	Cytochrome b6-f complex iron-sulphur subunit	Electron transport, Oxireductase	3	3	104.26
**A0A089VKJ7**	Photosystem I subunit III	Photosynthesis	5	4	104.08
**A0A089VKJ4**	`Photosystem I reaction centre subunit XI	Photosynthesis	9	8	104.06
**B5YMF5**	Acetyl-CoA carboxylase	ATP-binding	14	14	103.40
**Accession**	**Annotation**	**Go Process**	**Peptides**	**Unique**	**Significance (-logP)**
**KOT6D7**	Glyceraldehyde-3-phosphate dehydrogenase	Glycolysis, Oxireductase	15	14	103.28
**K0TIY5**	S-adenosylmethionine synthetic	ATP-binding, methionine adenosyltransferase activity	5	5	102.51
**B8BVI1**	Fucoxanthin-chlorophyll a-c binding protein, plastid	Photosynthesis	7	5	99.93
**B8CFG5**	Fucoxanthin chlorophyll a/c protein 4	Photosynthesis	8	7	99.36

**B8BUB2**	Probable 3-oxoacyl-reductase domain containing protein	Oxidoreductase	3	3	98.49
**K0SY05**	Glyceraldehyde-3-phosphate dehydrogenase	Glycolysis, Oxidoreductase	5	4	96.41
**B8BVT0**	Phosphate transport protein	Mitochondrial transport	5	3	96.17
**K3W4D8**	Class II fructose-bisphosphate aldolase	Glycolytic process	4	4	95.90
**A0A089X914**	ATP synthase subunit b, chloroplastic	ATP-synthesis	5	4	95.30
**K0RTZ4**	Tubulin beta chain	GTP-binding	4	4	95.07
**E7BWN1**	30S ribosomal protein S8, chloroplastic	Ribonucleoprotein, RNA-binding	2	2	94.13
**B8CE01**	Glutamate synthase	Oxidoreductase	7	6	94.02
**K0R9D2**	Ribosomal protein L19	Ribonucleoprotein	4	4	93.98
**B8BZG0**	CPSase	ATP-binding	17	16	93.96
**B8C239**	RL4e, ribosomal protein 4e 60S large ribosomal subunit	Ribonucleoprotein	3	3	93.96
**R1BJY8**	Quinone oxidoreductase	Oxidoreductase activity	4	4	93.36
**B8C590**	Ascorbate peroxide	Oxidoreductase, Peroxidase	2	2	92.62
**Accession**	**Annotation**	**Go Process**	**Peptides**	**Unique**	**Significance (-logP)**
**B8C2A9**	Malonyl CoA-acyl carrier protein transacylase	Acyltransferase	2	2	92.51
**K0TFW4**	Serine hydroxymethyltransferase	One-carbon metabolism	6	6	92.50
**B8C1P6**	Gdp-d-mannose 4,6-dehydratase	Lyase	4	4	92.27
**B8C0Z9**	Coatomer protein subunit beta2	Intracellular protein transport	4	4	92.07
**A0A089VP49**	ATP synthase subunit delta, chloroplastic	ATP synthesis, Hydrogen ion transport	5	5	91.01
**A0A089VKF6**	Photosystem II CP47 reaction centre protein	Photosynthesis	23	22	90.40

**B8BZT5**	Alpha enolase	Lyase	2	2	88.29
**B8BVM2**	Metalloprotease	Hydrolase, Metalloprotease	15	15	84.50
**K0SJ74**	Peptidyl-propyl cis-trans isomerase	Isomerase, Rotamase	4	3	82.12
**A0A089X950**	Cytochrome b6-f complex subunit 4	Electron transport	3	3	80.96
**B8BY77**	Rab-type small G protein	GTP-binding, Vesicle docking	5	2	79.57
**B8CEE0**	3-isopropylmalate dehydratase	Lyase	2	2	65.33
**B5YMV8**	Chaperone, heat shock protein 70	Stress response	2	2	61.36
**B8C1L0**	Cation transporting ATPase	Hydrolase	7	6	46.59
**B8BRY4**	Heat shock protein HslVU, ATPase	Stress response	3	3	29.53

[i] Protein inferred from homology.

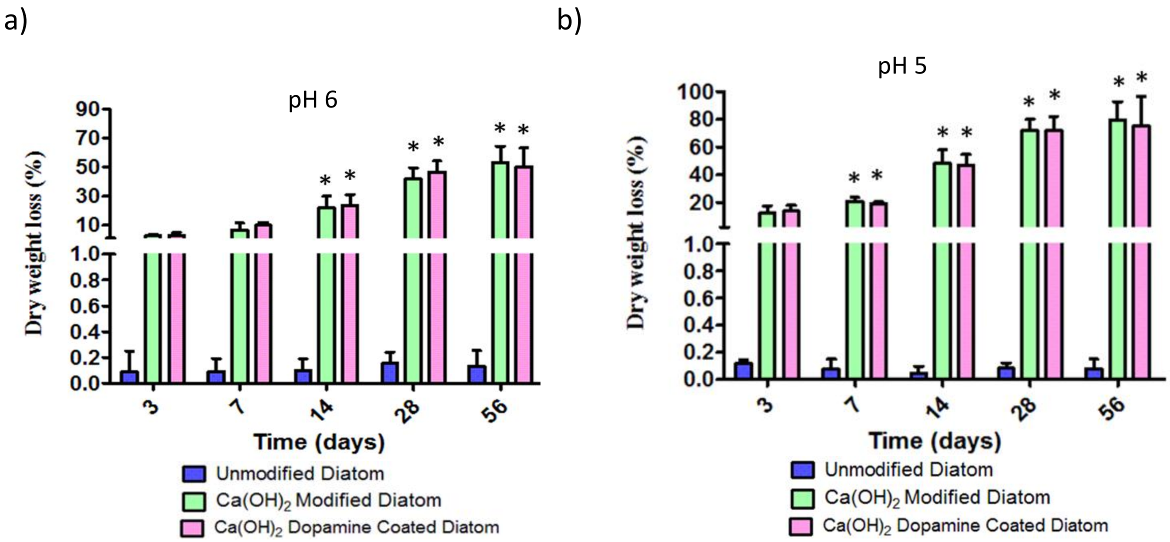

Figure S1. Degradation study of the diatoms. Dry weight loss rates (%) of the unmodified diatoms, $Ca(OH)_2$-modified diatoms, and $Ca(OH)_2$-modified diatoms coated with polydopamine at a) pH 6 and b) pH 5. Data are presented as the means ± S.E.M. (n=3). Two-way ANOVA followed by Bonferroni post-hoc analysis revealed significant differences at $^*P<0.05$.
